# Cognitive heterogeneity reveals molecular signatures of age-related impairment

**DOI:** 10.1093/pnasnexus/pgad101

**Published:** 2023-03-27

**Authors:** Sreemathi Logan, Matthew P Baier, Daniel B Owen, John Peasari, Kenneth L Jones, Rojina Ranjit, Hannah P Yarbrough, Anthony M Masingale, Suyesha Bhandari, Heather C Rice, Michael T Kinter, William E Sonntag

**Affiliations:** Center for Geroscience and Healthy Brain Aging, University of Oklahoma Health Sciences Center, Oklahoma City, OK 73104, USA; Department of Biochemistry and Molecular Biology, University of Oklahoma Health Sciences Center, Oklahoma City, OK 73104, USA; Department of Biochemistry and Molecular Biology, University of Oklahoma Health Sciences Center, Oklahoma City, OK 73104, USA; Department of Biochemistry and Molecular Biology, University of Oklahoma Health Sciences Center, Oklahoma City, OK 73104, USA; Center for Geroscience and Healthy Brain Aging, University of Oklahoma Health Sciences Center, Oklahoma City, OK 73104, USA; Department of Biochemistry and Molecular Biology, University of Oklahoma Health Sciences Center, Oklahoma City, OK 73104, USA; Department of Cell Biology, University of Oklahoma Health Sciences Center, Oklahoma City, OK 73104, USA; Department of Biochemistry and Molecular Biology, University of Oklahoma Health Sciences Center, Oklahoma City, OK 73104, USA; Department of Biochemistry and Molecular Biology, University of Oklahoma Health Sciences Center, Oklahoma City, OK 73104, USA; Department of Biochemistry and Molecular Biology, University of Oklahoma Health Sciences Center, Oklahoma City, OK 73104, USA; Department of Biochemistry and Molecular Biology, University of Oklahoma Health Sciences Center, Oklahoma City, OK 73104, USA; Center for Geroscience and Healthy Brain Aging, University of Oklahoma Health Sciences Center, Oklahoma City, OK 73104, USA; Department of Biochemistry and Molecular Biology, University of Oklahoma Health Sciences Center, Oklahoma City, OK 73104, USA; Aging and Metabolism Research Program, Oklahoma Medical Research Foundation, Oklahoma City, OK 73104, USA; Center for Geroscience and Healthy Brain Aging, University of Oklahoma Health Sciences Center, Oklahoma City, OK 73104, USA; Department of Biochemistry and Molecular Biology, University of Oklahoma Health Sciences Center, Oklahoma City, OK 73104, USA

**Keywords:** mitochondria, inflammation, heterogeneity, cognitive impairment, aging

## Abstract

The greatest risk factor for cognitive decline is aging. The biological mechanisms for this decline remain enigmatic due, in part, to the confounding of normal aging mechanisms and those that contribute to cognitive impairment. Importantly, many individuals exhibit impaired cognition in age, while some retain functionality despite their age. Here, we establish a behavioral testing paradigm to characterize age-related cognitive heterogeneity in inbred aged C57BL/6 mice and reliably separate animals into cognitively “intact” (resilient) and “impaired” subgroups using a high-resolution home-cage testing paradigm for spatial discrimination. RNA sequencing and subsequent pathway analyses of cognitively stratified mice revealed molecular signatures unique to cognitively impaired animals, including transcriptional down-regulation of genes involved in mitochondrial oxidative phosphorylation (OXPHOS) and sirtuin (*Sirt1* and *Sirt3*) expression in the hippocampus. Mitochondrial function assessed using high-resolution respirometry indicated a reduced OXPHOS coupling efficiency in cognitively impaired animals with subsequent hippocampal analyses revealing an increase in the oxidative damage marker (3-nitrotyrosine) and an up-regulation of antioxidant enzymes (Sod2, Sod1, Prdx6, etc.). Aged–impaired animals also showed increased levels of *IL-6* and *TNF-α* gene expression in the hippocampus and increased serum levels of proinflammatory cytokines, including IL-6. These results provide critical insight into the diversity of brain aging in inbred animals and reveal the unique mechanisms that separate cognitive resilience from cognitive impairment. Our data indicate the importance of cognitive stratification of aging animals to delineate the mechanisms underlying cognitive impairment and test the efficacy of therapeutic interventions.

**Significance Statement**

Cognitive decline with age is not uniform in mammals. Humans demonstrate substantial heterogeneity in age-related cognitive performance; some older subjects perform as well as young while others show substantial cognitive impairment. Our new methodology for behavioral testing in preclinical mouse models provides a statistically powerful approach to assess the causes of cognitive impairment. Our data delineate the role of mitochondrial metabolism, oxidative damage, and inflammatory signatures that predict impairment in aged mice. By stratifying mice into subgroups based on performance, we are able to identify target genes that predict impairment and potentially design intervention studies that mitigate cognitive impairment with age.

## Introduction

The etiology of age-related cognitive impairment and neurodegenerative diseases is one of the most compelling scientific and public health issues of our time. Cognitive decline with age in otherwise healthy subjects is an unanswered question that has an extremely high societal cost (1–8). Importantly, not all aging individuals exhibit cognitive impairment, and some individuals remain cognitively “resilient” and perform as well as young subjects until very late in life. The heterogeneity evident in humans with increasing age has generally been attributed to lifestyle, diet, and/or genetic factors, but the molecular mechanisms underlying these differences have been elusive. Moreover, while the concept of age-related cognitive heterogeneity has been generally well accepted in the human literature, this concept has not been rigorously assessed in preclinical models of aging. The etiology for the differences in cognitive function in aging individuals remains a key question for the field and research to establish the source and significance of these biological variations that are critical to advancing our understanding of the biological mechanisms that contribute to cognitive impairment and the development of effective interventions.

One of the major limitations in studying the biological mechanisms of cognitive heterogeneity is the lack of rigorously standardized cognitive testing paradigms for animal models that are capable of stratifying animals by behavioral ability. There are currently a wide range of cognitive tests used to assess differences in learning and memory, and, in many cases, these tests are specific to each laboratory. Consequently, relatively minor differences in experimental procedures between laboratories and even within the same lab are sufficient to drastically modify behavioral results. These, and other, issues make it difficult to reproduce results between laboratories and potentially mask the cognitive heterogeneity that naturally occurs with age. For these reasons, behavioral outcomes in aged animals are generally classified by mean performance while ignoring the high variability that exists within older animals. At a minimum, this approach not only reduces statistical power but also results in a missed opportunity to rigorously assess the cellular basis of cognitive resilience that occurs in older, cognitively intact animals. Accurately assessing cognitive heterogeneity with age and stratifying animals based on cognitive ability thus represent an inherently more powerful approach to address the molecular mechanisms of cognitive impairment and have the potential to reveal pathways that specifically contribute to cognitive dysfunction. To date, this type of analysis has rarely been pursued, especially in murine models of aging that are the most widely used preclinical model of cognitive aging.

Based on the compelling need to develop standardized testing protocols that can stratify aging mice, we developed behavioral testing paradigms that can separate aging animals into cognitively resilient and impaired subgroups. In this series of experiments, we demonstrate that aging animals can be reliably stratified and established that the increased cognitive heterogeneity with age is not the result of differences in movement or foraging behavior. In addition, we derive several new measures to compare young, aged–intact, and aged–impaired animals, including a measure of cognitive flexibility that can be assessed during reversal learning. Importantly, we find that RNA sequencing (RNAseq) analysis of cognitively stratified animals reveals specific changes in gene expression between these subgroups, including increases in oxidative damage and inflammatory cytokines and decreased mitochondrial efficiency. This approach to cognitive testing of aged animals is unique and highly reproducible and has the potential to standardize cognitive testing between laboratories. Further, we propose that age-specific stratification reveals relevant mechanisms that separate cognitive resilience from impairment.

## Results

### Cognitive heterogeneity increases with age

Heterogeneity in cognitive function in humans is well accepted yet has not been modeled efficiently in preclinical models to derive meaningful conclusions for successful interventions to target cognitive aging. We previously assessed aged mice using a high-resolution automated home-cage testing apparatus (PhenoTyper, Noldus) and reported impairment in cognitive function and an increase in variance in the aged group compared to young mice in several behavioral outcomes (9). Taking a systematic approach to unravel the source of this variation, we characterized cognitive function in aging C57BL/6N male mice in the PhenoTyper (Model 3000, Noldus Information Technology, Netherlands) as described previously (9–11). During testing, mice learned to enter one of three holes to receive a food pellet reward with performance (based on # of entries) recorded over a 90-h period during a 12-h day/night cycle. During acquisition (1–48 h), the correct entry choice was on the left and during reversal (49–89 h), and the correct entry choice was on the right. Corollary measurements of movement were recorded every 15th of a second and averaged on per hour performance. Initial learning rate and cognitive flexibility were derived from total entries during the first dark phase of the acquisition and reversal periods, respectively. Survival graphs were generated based on the percent of mice that reached 80% criterion (80% success rate in a moving window of the trailing 30 entries) as described in earlier reports (12).

**Fig. 1.**
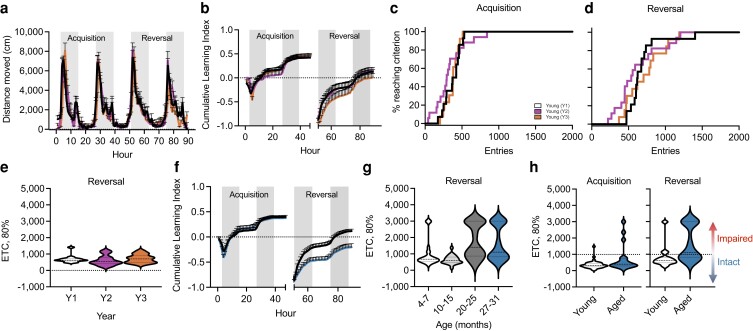
Assessment using the PhenoTyper demonstrating reproducibility over time and increasing cognitive heterogeneity with age. a) Circadian activity (distance moved) plotted per hour over a 90-h testing period in the PhenoTyper of young C57BL/6N male mice (4–7 months) cohorts assessed over a 3-year period (Y1-Y3). Shaded regions indicate dark periods of the light/dark cycle. b) Plotted cumulative learning index by year during initial learning (acquisition; 0–49 h) and reversal (49–89 h) phases show comparable performance across young cohorts. c and d) Percentage of mice reaching criterion based on correct entries during the acquisition (c) and reversal (d) phases of the behavioral task. e) Violin plots depicting the number of entries to achieve 80% criterion during the reversal phase were comparable among young cohorts across years. f) Plotted cumulative learning index of young (black) and aged (27–31 months; blue) animals depict age-related separation between groups during the reversal phase. g) Violin plots depicting the number of entries to achieve 80% criterion show increasing stratification within aged (20–25 months, 27–31 months; *n* = 27, *n* = 35) cohorts during the reversal phase compared to young (4–7 months; *n* = 24) and middle-aged (10–15 months; *n* = 22) mice. h) Violin plots depicting the number of entries required to achieve 80% criterion by young and aged (27–31 months) during the acquisition and reversal phases. Based on young performance metrics (90% confidence interval), aged animals were subsequently stratified into intact (<1,000 entries) and impaired (>1,000 entries) subgroups. For graphs a–e), colors represent the following: Y1 cohort (black/white, *n* = 14), Y2 cohort (purple, *n* = 17), and Y3 cohort (orange, *n* = 13). For graphs f) and h), colors represent the following: young (black, *n* = 40) and aged (blue, *n* = 35). Error bars depict the mean ± SEM.

Reference metrics for optimal performance in the PhenoTyper were developed using data from young (4–7 months) animals. Circadian activity (distance moved) for young male mice over the 90 h of testing in the PhenoTyper for the 3 years of testing (Y1–Y3) was superimposed (Fig. 1a) with the highest activity occurring during the dark phases (shaded regions). The cumulative learning index, calculated as the correct entries–incorrect entries/total entries, for young mice was also consistent over the 3 years of testing (Fig. 1b). Survival graphs for mice reaching criterion showed that young male mice performed consistently during both the acquisition (Fig. 1c) and reversal (Fig. 1d) phases with 97.5% of the mice reaching the 80% criterion. Young mice reached 80% criterion in less than 1,000 entries during the acquisition (Fig. S1a) and reversal (Fig. 1e) phases, which was reproducible over the 3-year testing period. Young animals were also capable of rapidly learning the reversal phase of the task, with 79% of this reference group reaching 80% success in under 1,000 entries (827 ± 104; mean ± SEM). We also measured hours to 80% criterion (HTC, 80%) for young animals which showed comparable performance over the 3 years of testing with most male mice reaching criterion in under 40 h during acquisition (Fig. S1b) and reversal (Fig. S1c). Performance of young animals in these measures was consistent across numerous cohorts of animals tested over the 3-year period, supporting the robustness of this reference metric.

Subsequent comparison of performance among young (4–7 months) and aged (27–31 months) mice showed an age-related decline in cumulative learning index during the reversal phase (Fig. 1f) as previously noted in Logan et al. (9). Further analysis of different age groups across the lifespan [young (4–7 months), middle-aged (10–15 months), and aged (20–25 and 27–31 months)] of male C57BL/6N mice revealed a marked heterogeneity in performance that increased with age (Fig. 1g). Performance in middle-aged and older animals during acquisition was comparable to young animals (Fig. S1c), but metrics for the reversal phase indicated increased heterogeneity. Moreover, a separation of groups into two distinct categories with independent performance characteristics was evident by 20–25 months of age (Fig. 1g). To define subgroups in the heterogeneous population of aged mice, we calculated the 90% confidence interval based on the entries to 80% criterion (ETC, 80%) of young animals during the reversal phase and applied that cutoff to categorize aged (27–31 months) animals as either cognitively “intact” (comparable to young) or cognitively “impaired” performers (Fig. 1h). Of the aged mice, 45.7% in the “intact” subgroup reached 80% criteria within 1,000 entries (intact ETC: 736.81 ± 37.78; *n* = 16), while 54.2% of mice in the “impaired” subgroup reached 80% criteria but required increased numbers of trials (impaired ETC: 2439.05 ± 181.51; *n* = 19) compared to younger groups (young ETC: 813.03 ± 105.92; *n* = 40).

### Behavioral characteristics of stratified aged–intact and aged–impaired subgroups

**Fig. 2.**
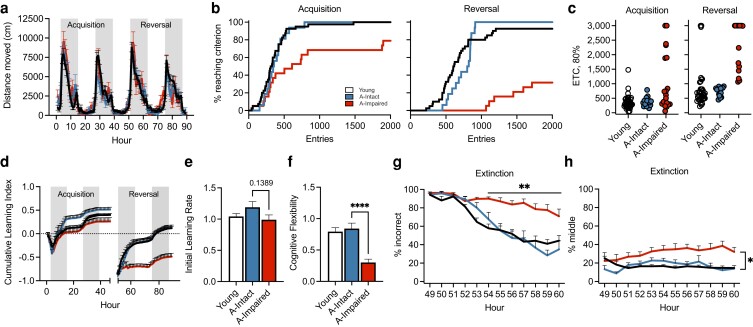
Performance of aged-stratified subgroups in the PhenoTyper reveals differences in cognitive ability. a) Circadian activity (distance moved) per hour during the PhenoTyper was comparable among young, aged–intact, and aged–impaired groups. Shaded gray bars indicate dark periods of the L:D cycle. b) Graphs detailing the percentage of mice reaching criterion indicates separation of the age–impaired group during the acquisition and reversal phase relative to young and aged–intact animals. c) Scatter plots depicting the number of entries required to achieve 80% criterion during the acquisition and reversal phases. d) Cumulative learning index during the acquisition and reversal phases. e) Bar plots show initial learning rate of aged–impaired animals relative to aged–intact and young animals during hours 3–12 of the acquisition phase. f) Bar plots depicting decreased cognitive flexibility of the aged–impaired animals relative to aged–intact group measured during hours 51–61 of the reversal phase (*P* < 0.0001). g) Percent of incorrect entries made by aged–impaired animals was significantly higher than young and aged–intact (*P* < 0.0001) groups between hours 54 and 60 during the extinction portion of the reversal phase. h) Percent of middle (incorrect) entries made by aged–impaired was significantly higher than that of young and aged–intact groups between hours 55 and 60 during the extinction portion of the reversal phase. For all graphs, colors represent the following: young (black/white, *n* = 41), aged–intact (blue, *n* = 16), and aged–impaired (red, *n* = 19). Error bars depict the mean ± SEM. Significance was tested using one-way (e and f) or two-way repeated measures ANOVA with post hoc Tukey comparisons (*P < 0.05; **P < 0.01; ****P < 0.0001).

After initial categorization based on the reversal criteria (1,000 entries), movement parameters were recalculated for the aged cohort. No differences were observed in circadian activity (Fig. 2a), body weights before and after testing (Fig. S2a), overall distance moved between light and dark phases (Fig. S2b), or average maximum segment velocity (Fig. S2c) with age or within the aged subgroups. Survival plots (based on ETC, 80%) were then recalculated for each of the aged subgroups for acquisition (Fig. 2b) and reversal phases (Fig. 2c). The data for aged–impaired mice demonstrated significant increases in the number of entries and decreases in the number of mice that reached 80% criterion compared to aged–intact and young groups during acquisition. This effect was more pronounced in the reversal phase. We found significant differences for the 80% ETC (Fig. 2d; log-rank, *P* < 0.01) and the 80% HTC (Fig. S2d; log-rank, *P* = 0.0095; Wilcoxon, *P* = 0.0123) between these groupings.

We then evaluated the cumulative learning index for acquisition and reversal phases which demonstrated a significant decline during the reversal stage in the aged–impaired mice (Fig. 2e). The independent learning index (correct entries–incorrect entries/total entries) was then calculated hourly for each animal and individual hour (Fig. S2e). Statistical significance was evaluated using the independent learning index values, and then the cumulative values were calculated. Significant differences were evident for the group main effect (*F*_2,73_ = 24.26, *P* < 0.0001) and the group × task interaction (*F*_2,6658_ = 58.03, *P* < 0.0001). Subsequent pairwise comparisons for the interaction effects indicated differences between the aged–intact and aged–impaired groups during acquisition [(*P* = 0.0023, Tukey honestly significant difference (HSD)] and between aged–impaired compared to the young and aged–intact groups during the reversal task (*P* < 0.0001, each). No significant differences were found between young and aged–intact groups during either acquisition or reversal phases. As expected, learning during the acquisition and reversal tasks occurred primarily during the dark phase of the light:dark (L:D) cycle while animals were awake and foraging for the food reward. Clear differences were apparent between the L:D phases during the study (*F*_1,6685_ = 37.8, *P* < 0.0001) with significant differences in the group × phase interaction (*F*_2,6685_ = 17.47, *P* < 0.0001). Additionally, we found differences between the L:D phases for the young and aged–intact groups (*P* < 0.0001, each), but not for the aged–impaired group (Fig. S2f; *P* = 0.88). Both young and aged–intact animals exhibited higher performance during the dark phase compared to impaired animals (*P* < 0.001); however, performance during the light phase was higher in aged–intact compared to impaired animals (*P* = 0.0008).

Initial learning rate was calculated during the 10 h (hours 3–12) of the first dark phase of acquisition, which showed no significant differences with either age or impairment (Fig. 2e; intact vs. impaired, *P* = 0.1389). Cognitive flexibility was then calculated over a 10-h period based on the ability to extinguish the learned behavior of the acquisition phase and relearn a new behavior. Using hours 51–61 of the first dark phase of the reversal task, data indicated that young and aged–intact animals exhibited similar cognitive flexibility (Fig. 2f). Animals categorized as aged–impaired exhibited a 62–64% decrease in cognitive flexibility compared to the other groups (*P* < 0.0001, each), reflecting both the slower rates of extinction and learning during the reversal task.

We further analyzed the rate at which animals learned the new task in the reversal phase by plotting the extinction curve of left entries, middle entries, and total errors (left + middle entries) during the first dark phase of the reversal. Analysis of extinction data after acquisition learning indicated a significant treatment × hour effect (*F*_18,572_ = 4.36, *P* < 0.0001) between groups based on performance during hours 51–60. While no differences were evident between young and aged–intact groups, impaired animals did not extinguish acquisition learning as rapidly when transitioned to the reversal phase. Percent errors (left + middle; Fig. 2g) and middle entries (Fig. 2h) were significantly higher in the aged–impaired group compared to aged–intact animals. Percent left entries trended higher in the aged–impaired group and significantly higher than aged–intact mice at hours 57 and 59 (Fig. S2g). This was confirmed by survival analysis of extinction curves between the three groups (log-rank, chi-square 11.96, *P* < 0.0025; Wilcoxon, chi-square 14.6, *P* < 0.0007). These data suggest that aged–impaired mice make more perseverating errors with increased middle entries and inability to extinguish a previously learned task that impairs relearning in the reversal phase.

We considered that differences in cognitive ability between groups may be related to differences in movement and therefore analyzed the activity of animals throughout the testing period. As expected, activity was highly dependent on the L:D cycle for all groups (*F*_1,6685_ = 1,551, *P* < 0.0001), though there were no significant differences between the group or group × phase interactions (Fig. S2b). We also considered that the food reward used in this test may affect motivation and would be correlated with activity. Differences in food consumption (pellets/hour) were evident based on the interaction between group, light phase, and task (*F*_2,6679_ = 10.8, *P* < 0.001). No group differences were found during the light phase of either the acquisition or reversal tasks for pellets dispensed (Fig. S2h, left panel). As expected, food consumption was reduced in the aged–impaired group compared to young and aged–intact animals (Fig. S2h, right panel; *P* < 0.001, each). These findings were consistent with the reduced learning during this period compared to young and aged–intact groups.

A major concern with any behavioral task that uses food reward is the potential for pronounced weight loss in underperforming animals. We analyzed prebehavior and postbehavior body weights and found that young, aged–intact, and aged–impaired animals lost 5.5 ± 0.7%, 5.3 ± 1.4%, and 6.6 ± 1.2% of their body weights during the study, respectively. No significant differences in prestudy and poststudy body weights (Fig. S2a) were found between any treatment groups.

### Cognitive stratification with age reveals a unique hippocampal transcriptional activity

**Fig. 3.**
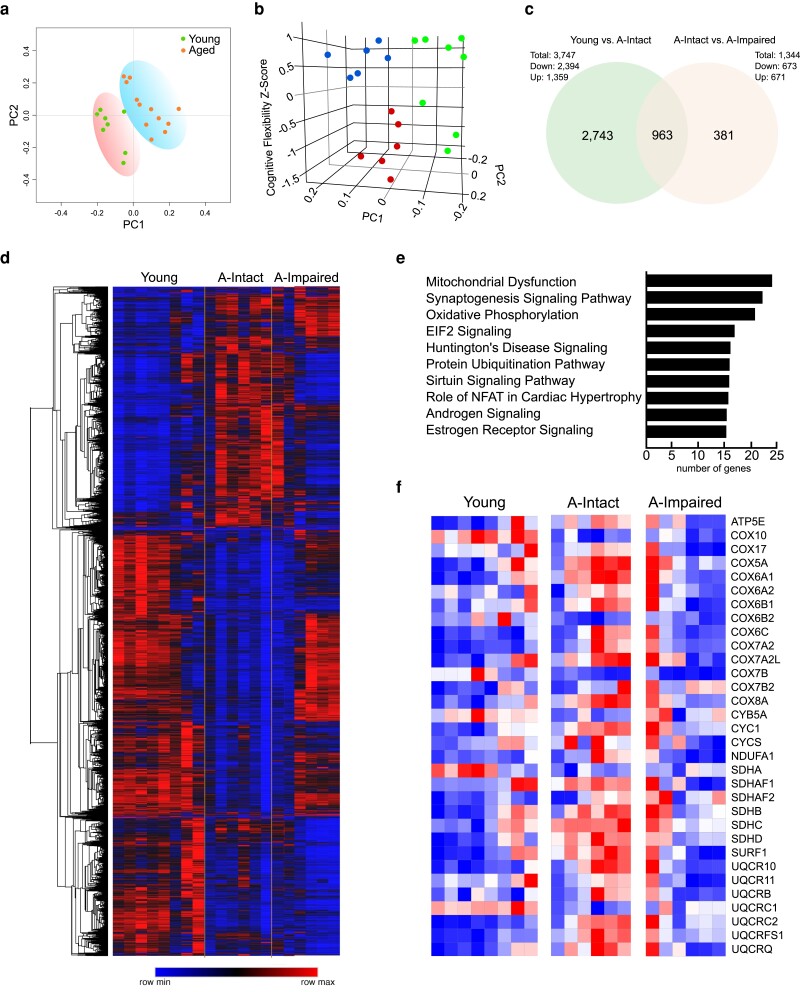
Cognitively stratified mice show distinct suppression of genes related to mitochondrial function in the hippocampus of aged–impaired mice. a) PCA of showing separation of young and aged mice. b) 3D PCA plot using cognitive flexibility *z*-scores as the third component depicting separation of young, aged–intact, and aged–impaired subgroups. c) Venn diagram illustrating all differentially expressed hippocampal genes from RNAseq analyses between young, aged–intact, and aged–impaired mice. d) Heatmap demonstrating the differential gene expression patterns with young, aged–intact, and aged–impaired hippocampi. e) IPA indicates the number of genes altered in specific pathways, with mitochondrial and OXPHOS genes significantly altered in the hippocampi of aged–impaired versus aged–intact mice. f) Heatmap depicting genes involved in mitochondrial OXPHOS with up-regulation of expression in aged–intact mice compared to young mice and dampened response in the aged–impaired compared to aged–intact mice.

To determine whether age-related cognitive impairment is associated with a unique molecular architecture, we isolated hippocampi in a subset of mice from each group of our study and analyzed transcriptional changes via RNAseq and ingenuity pathway analysis (IPA). Aged animals in each subcategory were selected from the intact (∼500–800 entries to criteria) or impaired (>2,000 entries to criteria) based on performance in the reversal task. Six biological replicates were used per group with 50 m reads per replicate. Principal component analysis (PCA) and transcriptome profiles showed separation of young and aged mice with increased variability apparent in the latter group (Fig. 3a). We further analyzed these data based on the stratified behavioral performance data (Fig. 3b). Utilizing the cognitive flexibility *z*-score as the third principal component along with PC1 and PC2, we were able to separate animals into three distinct groups (Fig. 3c).

RNAseq analysis revealed significant differences in gene expression profiles between aged–intact and impaired mice with a total of 963 genes significantly expressed with age (Fig. 3c). A total of 381 unique genes were differentially expressed in the aged–impaired animals relative to aged–intact. The number of genes significantly up-regulated or down-regulated with impairment between aged–intact and aged–impaired groups was then represented as a heatmap (Fig. 3d), and to identify pathways that were differentially activated within these subgroups, genes with significant changes were analyzed by IPA. Of the top three pathways, genes involved in oxidative phosphorylation (OXPHOS) were significantly down-regulated in aged–impaired animals compared to the aged–intact group (Fig. 3e). A total of 63 common OXPHOS genes were differentially expressed across the three groups, and 32 genes involved in mitochondrial OXPHOS showed significant down-regulation of gene expression in aged–impaired animals compared to the aged–intact group (Fig. 3f).

In additional analyses, sirtuins were investigated since they are a major component of metabolic regulation and regulate the activity of OXPHOS genes. RNAseq analyses revealed an up-regulation of each of the sirtuins genes (*Sirt1–Sirt7)* in aged–intact mice and a relative decline in sirtuin gene expression in impaired mice (Fig. S3a). We confirmed these finding by qPCR analyses for sirtuins gene expression. Both *Sirt1* (*P* = 0.0115) and *Sirt3* (*P* = 0.0113) were significantly down-regulated in the aged–impaired subgroup compared to aged–intact animals (Fig. S3b). No statistical differences were noted between aged–intact and young animals, while *Sirt3* expression was increased in the aged–intact group relative to young animals. These data are consistent with studies indicating a primary role for sirtuins in regulating metabolism in aging and cognitive function (13, 14) and a reduction in these factors leading to tissue dysfunction.

### Cognitive impairment in aged mice is marked by impaired mitochondrial function and increased oxidative stress

**Fig. 4.**
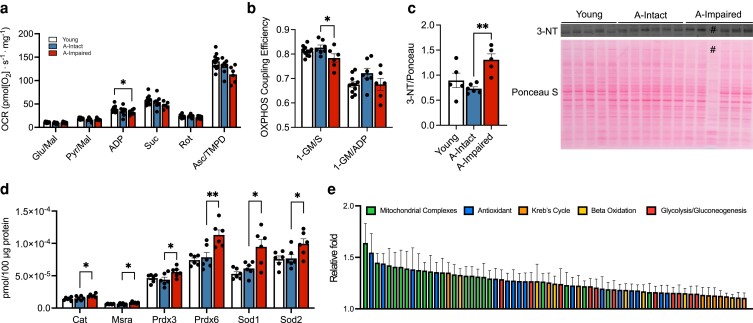
Cognitive stratification reveals mitochondrial dysfunction and readouts of oxidative stress in aged–impaired hippocampus. a) Bar plots depicting the OCR in hippocampal tissue extracted from young (*n* = 11), aged–intact (*n* = 7), and aged–impaired (*n* = 6) animals in response to the substrates glutamate/malate (Glu/Mal), pyruvate/malate (Pyr/Mal), ADP (2,500 *µ*m; *P* = 0.0138), succinate, rotenone, and ascorbate/TMPD. b) OXPHOS coupling efficiency (1-GM/S) as significantly reduced (*P* = 0.0297) in the aged–impaired group relative the aged–intact animals. c) Western blot quantification of the oxidative stress marker 3-NT normalized to total protein is significantly increased in aged–impaired animals (*n* = 5) compared to young (*n* = 5) and aged–intact (*n* = 6) groups (*P* = 0.003). *#* was excluded due to technical inconsistency in total protein levels. d) Bar graphs depicting quantification of the following antioxidant proteins with increased expression in the aged–impaired animals relative to the aged–intact group (*n* = 6/group): catalase (*P* = 0.0449), methionine sulfoxide reductase (*P* = 0.0173), peroxiredoxin 3 (*P* = 0.0429), peroxiredoxin 6 (*P* = 0.0037), superoxide dismutase 1 (*P* = 0.0105), and superoxide dismutase 2 (*P* = 0.0443). e) Bar graphs depicting the relative fold (aged–impaired over aged–intact) increases of mitochondrial complex (green), antioxidant (blue), Kreb's cycle (orange), β-oxidation (yellow), and glycolysis/gluconeogenesis (red) proteins quantified via targeted proteomic mass spectrometry analysis (see Tables S1–S6). For graphs a–d), colors represent the following: young (white), aged–intact (blue), and aged–impaired (red). Error bars depict the mean ± SEM. Significance was tested using one-way ANOVA (*P < 0.05; **P < 0.01).

We further assessed whether transcriptional down-regulation of mitochondrial OXPHOS genes are associated with a functional deficit in mitochondrial respiration in aged–impaired mice. We therefore measured mitochondrial respiration as well as OXPHOS coupling efficiency (efficiency of the electron transport chain to generate adenosine triphosphate (ATP)) using Oxygraph-2k (O2k) respirometry and fluorometry. No differences in oxygen consumption between groups were seen with complex I substrates, glutamate/malate. The oxygen consumption rate (OCR) was significantly reduced in the aged–impaired hippocampus compared to young controls with adenosine diphosphate (ADP; 2,500 *µ*m) as substrate (Fig. 4a). Response to *N*,*N*,*N*′,*N*′-tetramethyl-p-phenylenediamine (TMPD)/ascorbate increased complex IV-dependent OCR across all groups with comparable activity across all groups. We further evaluated OCR responses to sub-saturating levels of ADP (0.8–2,500 *µ*m), which showed significant reduction of ADP-induced OCR (Fig. S4a) in the aged–impaired group relative to young hippocampus at 1,250 and 2,500 *µ*m (*P* = 0.0197; *P* = 0.0138). No differences were noted in the OCR between aged–intact and aged–impaired groups. However, OXPHOS coupling efficiency (1-GM/S) was significantly reduced in the aged–impaired (Fig. 4b; *P* = 0.029) compared to aged–intact hippocampus. These data suggest that functional deficiencies in mitochondria may contribute to cognitive impairment in aging.

We then investigated whether the impairment in hippocampal mitochondrial OXPHOS coupling efficiency affected oxidative stress by measuring damage marker, 3-nitrotyrosine (3-NT). Levels of 3-NT were significantly increased in aged–impaired hippocampi compared to the aged–intact group (Fig. 4c; *P* = 0.003). Targeted protein analysis by mass spectrometry revealed increased antioxidant protein expression including peroxiredoxins (Prdx3 and Prdx6), superoxide dismutases (SODs) (Sod2 and Sod1), and catalase (Cat) in the impaired subgroup (Fig. 4d). Additionally, there was a general increase in expression of proteins involved in mitochondrial complexes, antioxidants, Kreb's cycle, β-oxidation, gluconeogenesis, and glycolysis in the aged–impaired hippocampi relative to the aged–intact group (Fig. 4e) and is detailed in supplementary information (Tables S1–S6). These data indicate that cognitive stratification of mice reveals distinct changes in mitochondrial gene and protein expression, impaired efficiency in mitochondrial function, and oxidative damage that correlates with impairment.

### Unique central and peripheral inflammatory signatures are associated with cognitive impairment in aging

**Fig. 5.**
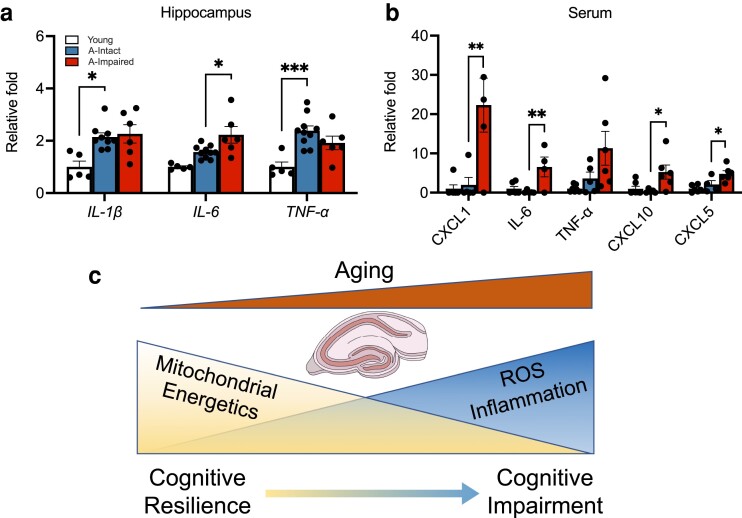
Increased inflammatory markers with impairment in aged mice. (a) Relative fold increases (compared to young, *n* = 5) in age-related hippocampal RNA expression of cytokines *IL-1β* (*P* = 0.017) and *TNF-α* (*P* = 0.0007) and the increase in *IL-6* (*P* = 0.021) between aged–impaired (*n* = 9) and aged–intact (*n* = 6) groups. b) Bar plots depicting the changes in serum cytokine levels of CXCL1 (*P* = 0.0068), IL-6 (*P* = 0.0086), TNF-α, CXCL10 (*P* = 0.0199), and CXCL5 (*P* = 0.0426) between the aged–impaired (*n* = 5) and aged–intact (*n* = 5) groups relative to young (*n* = 6). c) Schematic representing mitochondrial mechanisms, oxidative stress, and inflammatory markers underlying cognitive heterogeneity. For all graphs, colors represent the following: young (white), aged–intact (blue), aged–impaired (red). Error bars depict the mean ± SEM. Significance was tested using one-way ANOVA (*P < 0.05; **P < 0.01; ***P < 0.001).

Inflammation is a critical aspect of aging and age-related diseases and has been associated with cognitive impairment. To investigate whether inflammation is associated with cognitive impairment, we measured hippocampal expression of cytokines IL-1β, IL-6, and TNF-α. To avoid contribution of blood levels of cytokines, we measured mRNA levels of cytokines in the hippocampus by qRT-PCR (Fig. 5a). Levels of *IL-1β* (*P* = 0.017) and *TNF-α* (*P* = 0.0007) were increased with age but did not increase with impairment. However, levels of *IL-6* were significantly elevated in aged–impaired mice relative to the aged–intact group (*P* = 0.021). We further analyzed serum from young, aged–intact, and aged–impaired animals for inflammatory cytokines using multiplex immunoassays (Meso Scale Discovery). Serum levels of CXCL1 (*P* = 0.0068), IL-6 (*P* = 0.0086), CXCL10 (*P* = 0.0199), and CXCL5 (*P* = 0.0426) were significantly up-regulated in aged–impaired animals compared to the intact group, while levels of TNF-α trended higher in the impaired animals (Fig. 5b). These data suggest a critical link between inflammation, mitochondrial function, and cognitive impairment in a subgroup of aging animals (Fig. 5c).

## Discussion

Despite the heterogeneity of cognitive decline with age (15), studies of age-related cognitive decline in preclinical models routinely group animals based on their chronological age. Although this approach has increased our understanding of brain aging, no consensus has emerged related to the etiology of cognitive impairment or the mechanisms of age-related cognitive heterogeneity. In this study, we used inbred animals and capitalized on the age-related heterogeneity to reveal specific mechanisms associated with cognitive impairment. After development of specific performance metrics to stratify animals, we were able to reliably separate male mice into cognitively intact and impaired subgroups. This classification revealed clear differences in central metabolic pathways and peripheral inflammatory signatures that are differentially regulated with age and likely contribute to the heterogeneity of learning and memory deficits with age. Approximately 50% of the aged animals tested did not demonstrate inflammatory changes, mitochondrial dysfunction, and/or cognitive impairment, and inclusion of these animals would undoubtedly decrease the power to detect meaningful underpinnings of impairment with age. Thus, stratification by cognitive performance is inherently a more powerful approach to assess the effect of cognitive interventions. We propose that these new behavioral tests can be used to resolve the lack of uniformity in testing paradigms that exist between laboratories and reveal mechanisms that separate cognitive resilience and impairment in aged animals. This study utilized male mice for characterization of cognitive subgroups, largely due to the high *n* tested over the 3 years. Female mice are currently being assessed and analyzed using the same metrics, but due to relatively low numbers, threshold for cognitive stratification is still to be determined. Future studies will aim at cross comparisons of cognitively stratified male and female molecular and functional patterns that discern impairment or resilience in aging.

The concept of “cognitive resilience,” i.e. the ability to resist age-related cognitive impairment and neurodegeneration, has recently been recognized as an important aspect of cognitive aging research (16–23) but has been difficult to investigate. Importantly, the concepts of cognitive impairment and resilience result from categorizing individuals from a heterogeneous population, a finding that has been evident in the aging field for many years (15). In human studies, this information has been used to investigate the genetic components of “successful” aging, including diet, exercise, and specific drug interventions. In this study, we take advantage of the homogeneous genetic background in the inbred C57BL/6N mouse to control genetic variability and assess the noninherited biological mechanisms for cognitive resilience and impairment in aging. When animals were stratified by cognitive performance, gene expression signatures were evident that separated cognitive intact and impaired animals. Oxidative stress and antioxidant response genes were particularly affected, but the data indicate that the changes in gene expression are not a simple progression of changes that occur with age. Rather, it appears that there are unique gene expression changes between these two groups. These data are consistent with a model where cognitively impaired animals demonstrate a maladaptive response to oxidative damage. Furthermore, the data suggest that the diminished capacity to respond to age-associated oxidative stress is a seminal feature of cognitive impairment separate from “healthy” aging, resulting in the exacerbation of oxidative stress and the molecular/cellular mechanisms leading to cognitive impairment. Additionally, mitochondrial copy number and mitochondrial DNA (mtDNA) mutations have been shown to be increased with age and with skeletal muscle dysfunction (24). These deletion mutations have significant deleterious consequences on mitochondrial transcribed and miscoded ETC complex proteins that result in decreased efficiency of mitochondrial and tissue function. Whether the impairment in mitochondrial output in cognitively impaired animals is indicative of alterations in mtDNA mutations and copy number variations is an important area of investigation for future studies.

Cognitive heterogeneity in individuals may arise from many factors including genetic, environmental, or social factors that influence development of the brain and plasticity in response to the external environment as we age. Genetic contribution to learning and memory is well documented in Alzheimer's disease (AD) and other neurodegenerative disorders in humans and rodent models (25). Despite the strong association between amyloid load and AD, many aged human individuals remain resilient to amyloid-induced pathological consequences (26), suggesting that inherent processes of aging confer susceptibility or resilience to disease. One proteomics study demonstrated an enrichment of mitochondrial OXPHOS and reduction of glycolytic proteins as a marker for cognitive resilience in AD (27). Additionally, preservation of synaptic markers (28) and a reduction in inflammatory cytokines (29) are features associated with nondemented humans that show resilience to pathology. Thus, our results are indicative of poor resilience in aged cognitively impaired subjects and may be mediated through common mechanisms to drive cognitive trajectories in aging and AD. Additionally, genetically heterogeneous diversity outbred (DO) mice recapitulate the enhanced variability in multiple measures including cognitive function in aged mice (30). Thus, investigating cognitive trajectories in DO mice using the methodologies presented here could provide critical information of the interaction between basic aging processes and genetic diversity.

The interactions between psychosocial behaviors and learning are well known in the human and nonhuman primate literature (31, 32). In fact, early-life events, such as trauma or nutritional deprivation, can have long-lasting negative effects on cognitive function that manifest later in life. The effects of early-life stress on learning have been well documented in the human literature (33, 34) and in the preclinical literature (35–37) and are mediated, in part, through glucocorticoid actions (38). Both early-life psychosocial adversity and reduced caloric availability impair cognitive function later in life (39–42). Additionally, group-housed mice rapidly develop a social hierarchy within their home cage resulting in social stress and changes in immune responses in subsets of animals (43). Both male and female mice establish social hierarchies that have profound effects on psychosocial stress. The cognitive heterogeneity observed in this study of C57BL/6N inbred male mice, with the lack of genetic diversity, could result from social factors that drive a hierarchical organization of cage mates. Whether dominance and social hierarchy within littermates housed together influences cognitive trajectories in aging is unclear and warrants further investigation.

Activation of inflammatory pathways in the brain and in the peripheral system is a pathological outcome in age-associated neurodegenerative conditions and infection-induced neurological injury (44–46). Emerging data indicate that T cells release cytokines that have direct effects on multiple brain cell subtypes including astrocytes, microglia, and neurons/synapses and have a role in neurodegeneration in both AD and Parkinson's disease (46–50). Our data show an increase in circulating levels of inflammatory cytokines, while cognitively intact animals maintain levels comparable to young animals. Although the specific mechanisms for cognitive impairment remain unknown, it is of particular interest that levels and expression of IL-6 are increased in the impaired subgroup, both centrally and peripherally. IL-6 *trans*-signaling is a proinflammatory pathway known to induce reactive astrogliosis in the brain and is associated with the pathophysiology of central nervous system (CNS) injury, stroke, and neurodegenerative diseases such as AD and Parkinson's disease [reviewed by Garcia-Juarez and Camacho-Morales and Erta et al. (51, 52)]. Studies have shown that mitochondrial targeted antioxidants significantly reduce astrogliosis and proinflammatory cytokine production induced by manganese toxicity in cultured astrocytes (53), thereby linking mitochondrial antioxidant capacity to the inflammatory astrocyte phenotype. A probable hypothesis for why inflammation increases with age could be due to the increase in senescent cells both in the CNS and in the periphery. In fact, the accumulation of senescent cells has been reported in numerous tissues including the brain, fat, muscle, skin, bone marrow, etc. (54, 55). These cells are increased with age and in response to other damaging conditions including DNA damage, mitochondrial dysfunction, cell stress, and various oncogene mutations. Importantly, senescent-associated secretory products (SASP) secreted from these cells not only have important effects on neighboring cells but also circulate in the blood and influence distant tissue functions (56–62). Products of the SASP have been reported to include chemokines, cytokines, extracellular matrix proteases, remodeling factors, and bioactive lipids (57, 58, 63–65). The potential for the variability in the expression or timing of SASP-related products to drive inflammation and age-related cognitive impairment is currently under investigation.

Future studies on cell-specific transcriptional changes on cognitively stratified animals could yield further insight into the sources of cognitive heterogeneity with age. Additionally, studies aimed at investigating the genesis of inflammation, including senescence and SASP, and the interaction between inflammation and mitochondrial function both centrally and peripherally would be relevant to gain a better understanding of age-related loss of cognitive function and susceptibility to neurodegenerative disorders.

## Materials and methods

### Animals

All procedures were approved and conducted in accordance with the guidelines of the Institutional Animal Care and Use Committee at the University of Oklahoma Health Sciences Center (OUHSC). Male C57BL/6N mice of varying ages (4–7, 10–15, 20–25, and 27–31 months) were used for behavioral testing.

### Automated home-cage testing (PhenoTyper)

Spontaneous activity, acquisition (discrimination) learning, and reversal learning were assessed using an automated home-cage testing apparatus, PhenoTyper (Model 3000, Noldus Information Technology, Netherlands), as described previously (Maroteaux et al. 2012; Loos et al. 2014; Logan et al. 2018b). The following dependent variables to reach a specific success rate were calculated for both acquisition (discrimination) and reversal learning: percent of animals reaching criteria; ETC (80%), errors to criteria, time (hours) to criteria, cumulative learning index, and extinction curves (for details, see Supplementary Methods).

### Tissue harvest and processing

Following cognitive testing in the PhenoTyper, animals were euthanized, brains rapidly harvested, and hippocampi dissected and immediately processed for O2k respirometry or flash frozen in liquid nitrogen and stored at −80 °C until further use. Hippocampi were processed for RNA and cDNA as previously described (66).

### Library preparation and sequencing

Total RNA (200–500 ng) was used to prepare the Illumina HiSeq libraries [Laboratory for Molecular Biology and Cytometry Research (LMBCR, OUHSC)] according to the manufacturer's instructions for the Swift RNA kit (Swift Biosciences, Ann Arbor, MI, USA). Libraries were then sequenced using next-generation sequencing technology (Genomics and Sequencing Core Facility, OUHSC) using the Illumina NovaSeq 6000 platform with 50 m 2 × 150 bp reads/sample. Derived sequences were analyzed by applying a custom computational pipeline consisting of the open-source gSNAP (Wu and Nacu, 2010) and Cufflinks (Trapnell et al. 2010). R was used for alignment and discovery of differential gene expression (Baird et al. 2014).

### High-resolution respirometry (OROBOROS)

OCR and the rate of mitochondrial hydrogen peroxide production were simultaneously determined using the O2k (OROBOROS Instruments, Innsbruck, Austria) and O2k-Fluo LED2-Module Fluorescence-Sensor Green. Following rapid dissection, the dorsal hippocampus (2–3 mg) was suspended in ice cold Buffer X (7.23 mm K_2_EGTA, 2.77 mm CaK_2_EGTA, 20 mm imidazole, 0.5 mm DTT, 20 mm taurine, 5.7 mm ATP, 14.3 mm PCr, 6.56 mm MgCl_2_–6H2O, and 50 mm K-MES with a pH of 7.1) and permeabilized with saponin (50 *µ*g/ml) for 30 min followed by 3 × 5 min washes in ice-cold wash buffer Z [105 mm K-MES, 30 mm KCl, 10 mm K_2_HPO_4_, 5 mm MgCl_2_–6H2O, 0.5 mg/ml bovine serum albumin, 0.1 mm EGTA (pH 7.1)]. O2k measurements were obtained in buffer Z media at 37 °C containing 10 *µ*m Amplex UltraRed (Molecular Probes, Eugene, OR), 1 U/ml horseradish peroxidase, and 5 U/ml SOD. Rates of respiration were determined using sequential additions of substrates and inhibitors as follows: glutamate (10 mm), malate (2 mm), pyruvate (5 mm), ADP (5 mm), succinate (10 mm), rotenone (1 *µ*m), antimycin A (1 *µ*m), and TMPD (0.5 mm) immediately followed by ascorbate (5 mm). Antimycin A values were used to normalize respiration measurements to account for nonmitochondrial oxygen consumption. Data for OCR were normalized by milligrams of hippocampal wet weights.

### Inflammatory cytokine quantification

Levels of inflammatory cytokines (CXCL1, IL-6, TNF-α, CXCL10, and CXCL5) were measured using a U-PLEX assay (Meso Scale Diagnostics). Briefly, serum samples from young (*n* = 6), aged–intact (*n* = 5), and aged–impaired mice (*n* = *5*) were diluted in a 1:1 ratio using the diluent provided. Following coating of the U-PLEX plate with prepared linker-coupled capture antibodies, the assay was completed according to the manufacturer's instructions and quantified using a MESO QuickPlex SQ 120 reader (Meso Scale Diagnostics). Calibrators and samples were analyzed in duplicate and singlets, respectively.

### Statistical analyses

Statistical analyses were done using GraphPad Prism 9.4.0.673 and JMP v15.2.0 (SAS Inc.) software. Behavioral data were analyzed using an age × group × time repeated measures ANOVA using JMP as previously reported (9). Longitudinal measurements of interest for each mouse and their dependence of covariates were analyzed by fitting a linear mixed-effects model as implemented by the “lme” function in the R package “nmle.” The mixed-effects model extends the classical linear model (regression/ANOVA) by including random effects to accommodate for longitudinal intra-correlation and complex, nested, multilayer experimental design. Aged animals were stratified into intact and impaired subgroups based on a 90% confidence interval for the young reference group. All other molecular data were analyzed using a one-way ANOVA with Šidák's multiple comparisons test between young/aged–intact and aged–intact/aged–impaired groups unless otherwise noted. Data are represented as the mean ± SEM. Sample sizes are noted for each graph and significance noted with *P < 0.05; **P < 0.01; ***P < 0.001; ****P < 0.0001.

## Supplementary Material

pgad101_Supplementary_Data

## Data Availability

All data that support the findings of this study are available in this manuscript and the Supplementary material.
