# Supplementary material for: Cognitive heterogeneity reveals molecular signatures of age-related impairment
Source: PNAS Nexus. 2023 Mar 27;2(4):pgad101. doi: 10.1093/pnasnexus/pgad101 (PMC10118303; doi:10.1093/pnasnexus/pgad101)
Supplement: pgad101_Supplementary_Data [file pgad101_supplementary_data.pdf]

Supporting Information for
Cognitive Heterogeneity Reveals Molecular Signatures of Age-Related Impairment

Sreemathi Logan[1,2]*, Matthew P. Baier[2], Daniel B. Owen[2], John Peasari[1,2], Kenneth L. Jones[3], Rojina Ranjit[2], Hannah P. Yarbrough[2], Anthony M. Masingale[2], Suyesha Bhandari[2], Heather C. Rice[1,2], Michael T. Kinter[4], William E. Sonntag[1,2]

[1]Center for Geroscience and Healthy Brain Aging

[2]Department of Biochemistry and Molecular Biology, University of Oklahoma Health Sciences Center, Oklahoma City, OK

[3]Department of Cell Biology, University of Oklahoma Health Sciences Center, Oklahoma City, OK

[4]Aging & Metabolism Research Program, Oklahoma Medical Research Foundation, Oklahoma City, Oklahoma 73104

*Corresponding author: Sreemathi Logan, PhD
Email: Sreemathi-Logan@ouhsc.edu

This PDF file includes:

Supporting text
Figures S1 to S4
Tables S1 to S6
SI References

Supporting Information Text

Methods

Animals

Male mice were obtained from NIH rodent colony and housed in the specific pathogen free (including helicobacter and parvovirus) Rodent Facility at OUHSC. Mice were housed (3–4 per cage) in Allentown XJ cages with Anderson's Enrich-o-cob bedding (Maumee, OH) and maintained in a 12-h light/12-h dark cycle at 21°C with ad libitum access to standard irradiated bacteria-free rodent chow (5053 Pico Lab, Purina Mills, Richmond, IN) and reverse osmosis filtered water.

Automated Home-Cage Testing (PhenoTyper)

Activity of the mice in specifically designated areas of the PhenoTyper were recorded using EthoVision XT 14 software (Noldus Information Technology, Wageningen, The Netherlands) as previously reported (1). Mice were placed individually in the PhenoTyper for a 6-hour adaptation period and beginning at 1600 h on day 1, the activity of the animals was continuously recorded for 90 hours. All movements were recorded with an infrared-sensitive video camera above the arena using the X-Y coordinates of the center of gravity of each animal. These data were sampled at a rate of 15 frames per second (fps) and processed by Lowess smoothing using EthoVision (2, 3). The cages were transparent plastic (L x W x H =30 x 30 x 35 cm) and bedding was cellulose-free paper (Pure-o'Cel; The Andersons, Maumee, OH). Body weights of mice were measured immediately before the initiation of the study and immediately after completing the behavioral tasks. Behavior was tracked by video recording and the food dispenser was triggered by specific behaviors of the mouse (2). Water was available *ad libitum*.

During the acquisition phase of the test, mice were required to pass through the left entrance of the CognitionWall during initial discrimination to obtain a food reward (Dustless Precision Rodent Pellets, F05684, Bio-Serv, Flemington, NJ). After 49 hours of discrimination (acquisition) learning, the task was modified and the correct response was changed to the right entry, requiring the animal to extinguish the previous learning and acquire a new response, again rewarded using a FR5

schedule. The data were exported from EthoVision as a text file and then processed using Python scripts. Success rates for both acquisition and reversal learning were calculated and defined as the percentage of correct entries of the trailing 30 entries. The percentage of correct entries made in a moving window of the trailing 30 entries was calculated and trials to reach an 80% success rate determined. Thus, when the mouse achieved 80% correct entries within that window, the criteria was met, and the number of entries was plotted against the percentage of mice that achieved criteria for the group. The Independent Leaning Index was also calculated per hour based on correct entries minus the incorrect entries divided by the total number of entries and plotted as a cumulative learning index for acquisition and reversal learning. Cognitive flexibility was calculated as correct entries minus incorrect entries divided by the total number of entries during the first dark phase of reversal learning between hours 51 and 61 after initiation of the experiment. Extinction of learned behavior was calculated based on entries into left and middle entries (% incorrect) during the first dark phase of the reversal learning. It should be noted that animals are removed from the study if they received fewer than 10 pellet drops during the acquisition task or due to technical issues with pellet dispensers. This exclusion represented <10% of all mice in our study, irrespective of age. Following behavior testing, all mice were returned to group housing with chow food for one week before harvesting for tissue.

RNA/cDNA Preparation and qRT-PCR

Total RNA was extracted using the RNeasy mini kit (Qiagen, Germantown, MD). cDNA was prepared from equal concentrations of total RNA (1.0 µg) using the High-Capacity RNA-to-cDNA™ Kit (Applied Biosystems, Foster City, CA). qRT-PCR was performed using the following gene-specific Taqman probes: *Gfap* (Mm01253033_m1), *Aldh1l1* (Mm03048957_m1), *Gapdh* (Mm99999915_g1) and *Hprt* (Mm03024075_m1) were used as housekeeping genes for normalization. Quantitative PCR and melt-curve analyses were performed using TaqMan® Universal PCR Master Mix with UNG (Applied Biosystems) and the QuantStudio 12K Flex Real-Time PCR System (ThermoFisher Scientific). Gene Expression data were calculated from 5-6

independent samples unless otherwise stated, each with two replicates and are presented relative to the expression of the geometric mean of the house keeping genes (mean ± SEM).

Library preparation and sequencing

Total RNA extracted from the hippocampi was suspended in RNase-free water and RNA purity and concentration was measured on an Agilent Bioanalyzer (Agilent Technologies., Palo Alto, CA) and HiSeq libraries were sequenced. Each read generated was mapped to the mouse genome (mm10) by gSNAP. In addition to the genome sequence, gSNAP also accounts for splice variations, and SNP variants from dbSNP, and uses these ancillary databases to assist in mapping highly polymorphic sample data to the monomorphic reference genome, thereby increasing the mapping accuracy. Subsequently, Cufflinks calculates the prevalence of transcripts from each known gene based on normalized read counts. For each gene, Cufflinks quantifies transcript levels in fragments per kilobase of exon per million mapped reads (FPKM). The FPKM reflects the molar concentration of a transcript in the starting sample by normalizing for gene length and for the total read number in the sample. This allows for comparison of transcript levels both within and between experiments. From this information, we then determined significant gene expression using ANOVA in R. Once a gene list had been formed, we utilized pathway and gene set enrichment analyses to identify pathways of interest that may be modified. Genes significant at an FDR < 0.05 were submitted to pathway analysis using Ingenuity Pathway Analysis (Qiagen, Germantown, MD) to identify pathways of interest that were modified by age and cognitive status.

Targeted Quantitative Proteomics

Lysates (100 µg protein; n = 6/group) were run on an SDS gel for analysis as previously described (4). Each gel lane was cut as a complete sample, then divided into smaller pieces and washed/destained. The proteins were reduced with DTT and alkylated with iodoacetamide. Samples were then washed with ethanol and bicarbonate and digested with 1 µg of trypsin overnight at room temperature. The peptides produced were extracted from the gel, the extract evaporated to dryness, and reconstituted in 1% acetic acid for analysis. The digest samples were

injected in 5μl aliquots and quantified using an QEx orbitrap and TSQ systems. A BSA internal standard was added for quantification, and the mass spectrometer was operated in selected reaction monitoring (SRM) mode to analyze two peptides per protein. Data were analyzed using the program SkyLine, and the response for each protein was calculated as the geometric mean of the two-peptide area normalized to the total ion count (TIC) (5). The principal component analysis (PCA) plot was generated using ClustVis with default settings (Row scaling = unit variance scaling, PCA method = SVD with imputation, clustering distance for rows = correlation, clustering method for rows = average, tree ordering for rows = tightest cluster first) (6). Normalized values of each individual protein were analyzed via one-way ANOVA across young/aged-intact and aged-intact/aged-impaired groups.

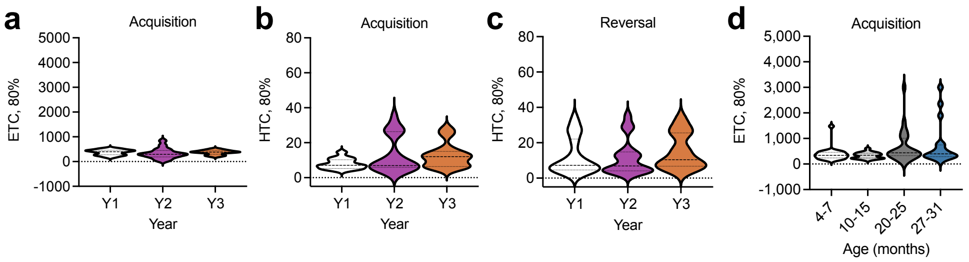

Fig. S1. Assessment using PhenoTyper demonstrates increasing cognitive heterogeneity with age.

(a) Violin plots depicting the number of entries to achieve 80% criterion during the acquisition phase of the PhenoTyper were comparable among young cohorts across a 3-year period.

(b-c) Hours required to achieve 80% criterion during the acquisition (b) and reversal (c) phases of the PhenoTyper were comparable among young cohorts across years tested.

(d) The number of entries to achieve 80% criterion show increasing stratification within aged (20-25 mo, 27-31 mo; n = 27, n = 35) cohorts during the acquisition phase compared to young (4-7 mo; n = 24) and middle aged (10-15 mo; n = 22) mice.

For graphs (a-d), colors represent the following: Y1 cohort (black, n = 14), Y2 cohort (purple, n = 17), Y3 cohort (orange, n = 13).

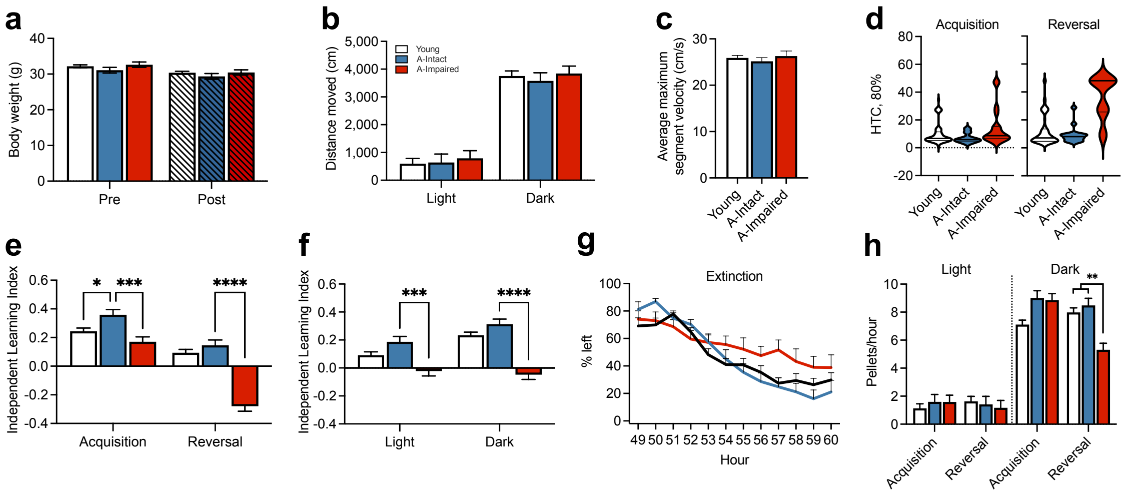

Fig. S2. Performance of aged-stratified subgroups in the PhenoTyper reveal differences in cognitive ability.

(a) Bar plots depicting the body weights of experimental cohorts were comparable during behavioral testing. Shaded bars indicate post-PhenoTyper weights.

(b) The total distance moved between cohorts were comparable during the light and dark phases of the PhenoTyper, respectively.

(c) Average maximum segment velocity in the PhenoTyper was comparable among groups.

(d) Violin plots depicting the hours required to achieve 80% criterion during the acquisition (left panel) and reversal (right panel) phases of the PhenoTyper.

(e) Plotted independent learning index during initial learning (acquisition; 0-49 h) and reversal (50-89 h) phases show a significant increase in performance by the aged-intact group relative to young (p = 0.0279) and aged-impaired (p = 0.0008) animals during the acquisition phase. Aged-impaired animals had a significant decrease (p < 0.0001) in performance relative to aged-intact animals during the reversal phase.

(f) The independent learning index indicates decreased performance in the aged-impaired group relative to aged-intact animals during both the light (p = 0.0002) and dark (p < 0.0001) phases of the L:D cycle.

(g) Plot depicting the percent of left entries were comparable among groups during the extinction portion of the reversal phase.

(h) Bar plot depicting the distribution of pellets per hour of the aged-impaired group was decreased compared to young and aged-intact animals (p = 0.0067) during the dark portion of the reversal phase.

For all graphs, colors represent the following: young (black, n = 41), aged intact (blue, n = 16), aged impaired (red, n = 19). Error bars depict the mean ± SEM. Significance was tested using two-way ANOVA (*p < 0.05, **p < 0.01, ***p < 0.001, ****p < 0.0001)

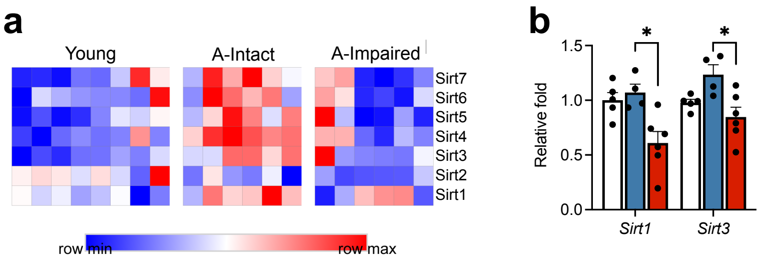

Fig. S3. Cognitively stratified mice show a distinct reduction in expression of genes related to mitochondrial function in the hippocampus of aged-impaired mice.

(a) Heat map depicting the differential expression of sirtuins among groups.
(b) Bar plots depicting the decrease in relative fold (compared to young; black, $n = 5$) expression of *Sirt1* ($p = 0.0115$) and *Sirt3* ($p = 0.0113$) between aged-impaired (red, $n = 6$) and aged-intact (blue, $n = 4$) groups.

Error bars depict the mean ± SEM. Significance was tested using one-way ANOVA (*$p < 0.05$).

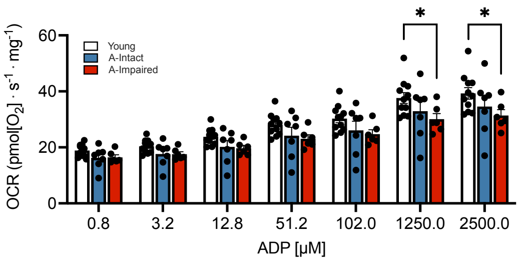

Fig. S4. Cognitive stratification reveals mitochondrial dysfunction and increased oxidative stress in aged-impaired hippocampus.

Oxygen consumption rate in hippocampal tissue extracted from young (black, n = 11), aged-intact (blue, n = 7), and aged-impaired (red, n = 6) animals in response to increasing concentrations of ADP. Age-related declines in OCR were detected at 1250 µM (p = 0.0197) and 2500 µM (p = 0.0138) concentrations.

Error bars depict the mean ± SEM. Significance was tested using two-way ANOVA (*p < 0.05).

Table S1. Mass spectrometry analysis listing abundance of targeted antioxidant proteins.

Antioxidant	young		aged intact		aged impaired	
	values x 10^{-5}					
**Protein**	**mean**	**SEM**	**mean**	**SEM**	**mean**	**SEM**
Akr1b1	2.93	0.17	3.22	0.29	3.61	0.23
Alb-m	46179.25	8034.42	37607.81	4938.92	52032.19	7857.29
Aldh2	4.68	0.30	5.05	0.47	5.48	0.20
Cat	1.42	0.10	1.51	0.14	***1.93**	0.11
Cryab	0.34	0.05	0.50	0.09	0.61	0.09
Gpx1	0.48	0.06	0.59	0.08	0.72	0.05
Gpx4	1.70	0.09	2.18	0.29	2.13	0.23
Gsr	1.15	0.05	1.30	0.12	1.40	0.10
Gsta3	4.47	0.36	4.68	0.38	5.94	0.37
Gstm1	33.15	1.80	36.40	3.58	44.67	3.07
Gstp1	10.60	0.51	13.62	1.61	16.30	1.56
Hsp90b1	9.55	0.56	9.42	0.74	9.59	0.52
Hspa1a	56.56	2.67	54.35	4.72	62.23	3.61
Hspa5	5.33	0.40	5.02	0.55	5.69	0.42
Hspa9	6.05	0.21	5.73	0.55	6.66	0.40
Lonp1	2.04	0.08	2.23	0.18	2.35	0.16
Lonp2	0.08	0.00	0.08	0.01	0.09	0.01
Msra	0.62	0.03	0.60	0.06	***0.82**	0.06
Phb	7.17	0.31	7.39	0.67	8.56	0.53
Phb2	9.90	0.71	10.20	0.75	11.68	1.14
Prdx1	6.99	0.41	8.49	0.85	10.13	0.83
Prdx2	13.82	1.16	14.35	1.06	17.02	0.75
Prdx3	4.63	0.26	4.39	0.39	***5.58**	0.32
Prdx5	8.58	0.74	10.19	1.55	12.78	1.60
Prdx6	7.42	0.32	7.85	0.75	****11.31**	0.77
Sod1	5.30	0.33	6.13	0.44	***9.47**	1.13
Sod2	7.57	0.49	7.69	0.60	***9.94**	0.76
Txn1	3.94	0.32	4.34	0.51	5.53	0.45
Txnrd1	1.28	0.04	1.40	0.11	1.60	0.09

Significant differences between aged intact and aged impaired groups are represented with an asterisk (*p < 0.05, **p < 0.01).

Table S2. Mass spectrometry analysis listing abundance of targeted complex 1 proteins.

Complex 1	young		aged intact		aged impaired	
	values x 10^{-5}					
Protein	mean	SEM	mean	SEM	mean	SEM
Ndufa1	1.11	0.07	1.32	0.15	1.41	0.15
Ndufa2	2.81	0.30	3.44	0.31	4.15	0.45
Ndufa3	3.00	0.17	3.87	0.56	4.04	0.37
Ndufa4	18.57	1.51	22.71	3.25	26.76	3.12
Ndufa5	2.47	0.23	3.08	0.43	3.65	0.37
Ndufa6	5.44	0.55	6.61	1.04	8.24	0.99
Ndufa7	1.72	0.14	2.00	0.22	2.71	0.27
Ndufa8	5.27	0.35	5.92	0.66	7.29	0.73
Ndufa9	8.28	0.54	8.06	0.35	9.60	0.54
Ndufa10	16.70	0.60	16.68	0.82	18.18	0.76
Ndufa11	2.83	0.28	3.11	0.42	4.07	0.41
Ndufa12	2.43	0.22	2.59	0.49	3.46	0.25
Ndufa13	10.20	1.05	10.97	1.96	14.15	1.62
Ndufb3	4.69	0.48	5.70	0.80	7.18	0.83
Ndufb4	1.46	0.08	1.73	0.27	2.13	0.23
Ndufb5	4.67	0.61	5.69	1.10	7.27	1.06
Ndufb6	2.26	0.18	2.67	0.38	3.47	0.38
Ndufb7	3.58	0.29	4.11	0.49	5.79	0.67
Ndufb8	4.01	0.24	4.92	0.55	6.08	0.54
Ndufb9	3.29	0.18	3.68	0.29	****5.05**	0.29
Ndufb10	8.64	0.56	10.21	0.88	***12.92**	0.78
Ndufb11	4.27	0.35	4.96	0.81	7.05	0.82
Ndufc2	4.16	0.42	5.11	0.64	6.24	0.61
Nd1	4.94	0.22	5.15	0.38	5.73	0.42
Nd2	0.93	0.04	1.03	0.06	1.07	0.06
Nd3	4.73	0.18	5.22	0.45	5.81	0.37
Nd4	2.37	0.12	2.41	0.19	2.84	0.21
Nd5	0.78	0.04	0.79	0.06	0.87	0.06
Ndufs1	48.29	1.94	53.62	4.60	55.87	3.27
Ndufs2	14.27	0.64	14.37	0.84	16.96	0.88
Ndufs3	25.46	1.55	25.46	1.73	30.01	1.39
Ndufs4	6.97	0.68	8.15	1.17	9.52	0.98
Ndufs5	4.93	0.42	5.80	0.70	***8.17**	0.81
Ndufs6	2.15	0.22	2.38	0.32	3.27	0.38

Ndufs7	4.23	0.32	4.85	0.51	5.93	0.41
Ndufs8	9.95	0.68	10.11	0.74	***13.28**	0.73
Ndufv1	25.44	0.91	27.31	2.05	30.19	1.56
Ndufv2	7.57	0.53	7.78	0.51	****11.27**	0.70

Significant differences between aged intact and aged impaired groups are represented with an asterisk (*p < 0.05, **p < 0.01).

Table S3. Mass spectrometry analysis listing abundance of targeted complexes 3, 4, and 5 proteins.

**Complexes 3, 4, 5**	**young**		**aged intact**		**aged impaired**	
	**values x 10^{-5}**					
**Protein**	**mean**	**SEM**	**mean**	**SEM**	**mean**	**SEM**
Cyc1	10.57	0.59	11.53	0.88	***15.24**	1.03
Cytb	3.17	0.15	3.08	0.25	3.59	0.26
Uqcrc1	12.53	0.63	12.67	1.09	13.58	0.69
Uqcrc2	11.72	1.19	12.17	1.36	11.85	0.98
Uqcr10	2.36	0.30	2.72	0.43	3.37	0.42
Uqcr11	0.54	0.06	0.62	0.11	0.84	0.10
Uqcrb	0.39	0.03	0.44	0.08	0.58	0.07
Uqcrfs1	6.20	0.45	6.01	0.56	7.61	0.35
Uqcrh	0.66	0.07	0.68	0.10	***1.12**	0.13
Uqcrq	3.59	0.33	4.36	0.65	5.41	0.71
Atp5a1	140.14	4.13	145.59	12.10	163.54	10.35
Atp5b	126.21	4.87	125.91	9.63	140.47	8.72
Atp5c1	26.17	1.28	25.16	1.98	30.86	2.25
Atp5d	6.11	0.70	7.38	1.31	8.26	1.13
Atp5e	1.09	0.19	1.28	0.22	1.76	0.29
Atp5o	13.68	1.16	14.26	1.20	17.77	1.23
Atp5pb	1.96	0.15	1.89	0.16	***2.45**	0.14
Atp5h	4.68	0.37	4.84	0.52	***6.39**	0.43
Atp5k	1.08	0.10	1.37	0.26	1.65	0.26
Atp5md	0.52	0.03	0.55	0.04	0.64	0.07
Atpl	5.14	0.56	5.71	0.93	6.58	0.76
Atp5j2	20.23	2.34	23.26	3.79	26.73	3.15
Atp5j2	0.41	0.05	0.38	0.03	0.53	0.06
Atp8-mt	1.99	0.19	2.65	0.36	2.66	0.36
Cox1	3.65	0.17	3.58	0.26	4.20	0.29
Co2-mt	53.09	4.32	56.40	4.74	68.85	2.86
Cox4i1	33.27	3.03	37.70	5.09	47.50	5.15
Cox5a	7.65	0.72	8.89	1.31	11.04	1.42
Cox5b	3.92	0.38	4.58	0.66	6.22	0.79
Cox6b1	2.96	0.28	3.51	0.41	4.76	0.54
Cox6c	6.58	0.95	7.84	1.31	9.21	1.26
Cox7a2	1.16	0.08	1.50	0.16	1.66	0.21

Significant differences between aged intact and aged impaired groups are represented with an asterisk (*$p < 0.05$).

Table S4. Mass spectrometry analysis listing abundance of targeted proteins in the Kreb's cycle.

**Kreb's cycle**	**young**		**aged intact**		**aged impaired**	
	**values x 10^{-5}**					
**Protein**	**mean**	**SEM**	**mean**	**SEM**	**mean**	**SEM**
Aco2	34.09	1.24	35.81	2.98	39.58	2.94
Cs	62.27	1.84	64.26	3.36	68.27	2.84
Dlat	50.04	2.00	52.35	3.45	57.97	2.47
Dld	9.15	0.76	8.78	0.83	10.98	0.76
Dlst	28.56	1.12	29.87	2.08	33.17	1.50
Fh1	3.33	0.12	3.27	0.20	3.67	0.10
Glud1	18.77	0.97	21.32	1.83	23.33	0.93
Idh1	3.53	0.21	4.04	0.34	3.96	0.20
Idh2	3.59	0.20	3.85	0.36	3.93	0.09
Idh3a	16.91	0.38	17.18	0.81	18.40	1.05
Idh3b	9.68	0.34	9.15	0.48	10.32	0.45
Idh3g	10.43	0.31	10.36	0.65	10.87	0.61
Mdh1	54.43	2.47	58.03	4.50	65.96	4.16
Mdh2	37.12	1.73	37.20	2.11	42.14	2.80
Ogdh	12.97	0.46	14.39	1.19	15.13	0.89
Pdha	22.37	0.89	24.01	1.53	25.22	1.22
Pdhb	29.89	1.60	31.85	2.36	35.97	2.40
Pdk1	1.39	0.05	1.39	0.08	1.46	0.06
Pdk2	0.94	0.03	0.98	0.06	1.01	0.04
Sdha	6.45	0.16	7.09	0.55	7.67	0.33
Sdhb	5.30	0.20	5.53	0.38	****7.48**	0.54
Sdhc	0.46	0.03	0.51	0.06	0.64	0.05
Sucla2	12.33	0.36	13.18	1.20	13.48	0.57
Suclg1	2.58	0.14	2.67	0.20	3.27	0.27

Significant differences between aged intact and aged impaired groups are represented with an asterisk (**p < 0.01).

Table S5. Mass spectrometry analysis listing abundance of targeted proteins of beta oxidation.

**Beta Oxidation**	**young**		**aged intact**		**aged impaired**	
	**values x 10^{-5}**					
**Protein**	**mean**	**SEM**	**mean**	**SEM**	**mean**	**SEM**
Abcd3	1.10	0.04	1.26	0.11	1.23	0.05
Acaa1a/b	0.42	0.01	0.46	0.04	0.48	0.02
Acaa2	1.11	0.10	1.10	0.11	1.05	0.05
Acadl	0.75	0.03	0.76	0.04	0.85	0.02
Acadm	1.16	0.07	1.24	0.10	1.23	0.04
Acads	0.77	0.04	0.83	0.09	0.90	0.02
Acadvl	0.66	0.06	0.83	0.08	0.80	0.05
Acot13	9.36	0.88	11.48	1.81	14.57	1.90
Ascl1	1.04	0.05	1.13	0.11	1.20	0.06
Bdh1	7.84	0.33	8.32	0.64	***10.37**	0.60
Cpt1a	0.74	0.05	0.72	0.07	0.73	0.04
Cpt2	0.94	0.04	1.08	0.10	1.12	0.12
Crat	0.52	0.01	0.57	0.04	***0.67**	0.03
Decr1	1.69	0.08	1.74	0.13	2.01	0.08
Ech1	1.48	0.05	1.75	0.18	1.80	0.11
Echs1	1.78	0.08	1.52	0.27	2.03	0.09
Eci1	0.65	0.03	0.61	0.05	0.71	0.04
Eci2	3.94	0.23	3.70	0.41	4.65	0.34
Etfa	6.06	0.30	5.98	0.53	6.59	0.41
Etfb	2.30	0.16	2.28	0.19	2.59	0.13
Etfdh	1.81	0.07	1.87	0.13	2.05	0.12
Fabp3	9.47	0.55	11.95	1.71	14.03	1.67
Fabp4	1.11	0.06	1.13	0.07	1.20	0.07
Glud1	18.77	0.97	21.32	1.83	23.33	0.93
Hadh	8.90	0.43	9.22	0.81	9.91	0.54
Hadha	2.72	0.17	2.98	0.26	3.00	0.22
Hadhb	2.30	0.09	2.46	0.20	2.49	0.07
Hsd17b4	1.27	0.06	1.38	0.13	1.51	0.08
Pecr	0.54	0.02	0.52	0.12	0.53	0.09
Slc25a20	1.88	0.10	2.00	0.17	2.22	0.14

Significant differences between aged intact and aged impaired groups are represented with an asterisk (*$p < 0.05$).

Table S6. Mass spectrometry analysis listing abundance of targeted proteins in glycolysis and gluconeogenesis.

**Glycolysis/Gluconeogenesis**	**young**		**aged intact**		**aged impaired**	
	**values x 10^{-5}**					
**Protein**	**mean**	**SEM**	**mean**	**SEM**	**mean**	**SEM**
Aldoa	182.97	6.95	183.73	8.72	205.43	14.72
Aldoc	12.17	0.75	13.03	0.90	15.07	0.70
Eno1	162.16	4.64	174.78	12.98	193.07	9.42
Eno2	17.99	0.57	17.69	1.42	21.57	1.32
G6pd	1.09	0.03	1.38	0.14	1.44	0.08
Gapdh	433.78	21.73	469.80	35.81	517.61	42.18
Got1	17.20	0.68	18.07	1.18	20.88	1.38
Got2	50.59	1.89	49.76	2.78	57.86	2.84
Gpi1	12.06	0.53	12.89	1.15	15.11	0.92
Hk1	43.96	1.97	48.10	3.10	48.72	2.79
Hspd1	21.08	1.03	19.68	1.16	19.68	1.16
Ldha	53.80	3.10	52.38	3.50	60.15	3.76
Ldhb	69.96	2.24	78.58	6.21	87.53	4.60
Mpc1	2.69	0.18	3.14	0.28	3.89	0.46
Mpc2	1.26	0.05	1.47	0.18	1.66	0.19
Pcx	3.86	0.16	4.40	0.40	4.87	0.29
Pfkfb2	0.47	0.02	0.49	0.05	0.54	0.03
Pfkl	22.19	0.87	23.03	1.90	25.03	2.22
Pfkm	12.68	0.51	13.81	1.26	15.50	1.01
Pgam1	40.50	1.97	40.37	3.42	***52.02**	3.62
Pgk1	22.16	0.65	22.75	1.58	25.14	1.08
Pkm	183.57	5.74	202.75	15.79	220.09	12.16
Pybg	5.87	0.45	6.85	0.65	7.88	0.51
Pybm	2.07	0.24	2.63	0.20	3.00	0.16
Slc2a1	5.48	0.31	6.68	0.66	7.05	0.30
Taldo1	2.82	0.11	2.91	0.23	3.46	0.25
Tkt	10.90	0.93	13.04	1.09	14.53	0.93
Tpi1	44.44	2.38	43.18	4.39	54.78	3.60
Vdac1	25.71	1.75	23.68	1.66	28.58	1.95
Vdac2	66.60	3.74	69.87	5.70	80.28	5.53
Vdac3	9.23	0.59	8.93	0.68	10.25	0.66

Significant differences between aged intact and aged impaired groups are represented with an asterisk (*$p < 0.05$).

SI References

1. S. Logan *et al.*, Simultaneous assessment of cognitive function, circadian rhythm, and spontaneous activity in aging mice. *Geroscience* **40**, 123-137 (2018).
2. G. Maroteaux *et al.*, High-throughput phenotyping of avoidance learning in mice discriminates different genotypes and identifies a novel gene. *Genes Brain Behav* **11**, 772-784 (2012).
3. M. Loos *et al.*, Sheltering behavior and locomotor activity in 11 genetically diverse common inbred mouse strains using home-cage monitoring. *PLoS One* **9**, e108563 (2014).
4. C. S. Kinter *et al.*, A quantitative proteomic profile of the Nrf2-mediated antioxidant response of macrophages to oxidized LDL determined by multiplexed selected reaction monitoring. *PLoS One* **7**, e50016 (2012).
5. S. O. Deininger *et al.*, Normalization in MALDI-TOF imaging datasets of proteins: practical considerations. *Anal Bioanal Chem* **401**, 167-181 (2011).
6. T. Metsalu, J. Vilo, ClustVis: a web tool for visualizing clustering of multivariate data using Principal Component Analysis and heatmap. *Nucleic Acids Res* **43**, W566-570 (2015).
